# Diagnostic and prognostic potential of long non-coding RNA NORAD in patients with acute deep vein thrombosis and its role in endothelial cell function

**DOI:** 10.1186/s12959-023-00575-3

**Published:** 2024-01-02

**Authors:** Kun Zhou, Na Li, Jia Qi, Pingping Tu, Yan Yang, Hui Duan

**Affiliations:** 1 Department of Breast Thyroid Vascular Surgery, Taihe Hospital, Affiliated Hospital of Hubei University of Medicine, 442000 Shiyan, China; 2 Department of Hematology, Taihe Hospital, Affiliated Hospital of Hubei University of Medicine, 442000 Shiyan, China; 3 Department of Ophthalmology, Taihe Hospital, Affiliated Hospital of Hubei University of Medicine, 442000 Shiyan, China; 4 Department of Emergency, Taihe Hospital, Affiliated Hospital of Hubei University of Medicine, No.32, Renmin South Road, 442000 Shiyan, Huibei Province China

**Keywords:** NORAD/miR-93-5p axis, Deep venous thrombosis, Clinical value, Endothelial cell dysfunction

## Abstract

**Background:**

Deep venous thrombosis (DVT) is the common clinical cardiovascular disease, and easily develops into post-thrombotic syndrome (PTS). The study aimed to examine the clinical value of long non-coding RNA *NORAD* gene in the development of DVT and PTS. In vitro, the underlying mechanism was explored.

**Methods:**

Serum levels of lncRNA *NORAD* gene in 85 DVT cases and 85 healthy individuals were tested. The role of lncRNA *NORAD* gene in human umbilical vein endothelial cells (HUVECs) proliferation, migration and inflammation was examined. The candidate downstream target gene was predicted via bioinformatic analysis. Gene ontology (GO) analysis and Kyoto Encyclopedia of Genes and Genomes (KEGG) pathway enrichment analysis were done for the function annotation and pathway enrichment.

**Results:**

LncRNA *NORAD* gene was at high expression in the serum of DVT patients, it can distinguish DVT patients from healthy controls with the area under the curve of 0.919. Elevated expression of lncRNA *NORAD* gene in PTS patients was detected, DVT cases with high expression of lncRNA *NORAD* gene were more susceptible to PTS. LncRNA *NORAD* gene knockdown promoted HUVECs’ proliferation, migration while suppressing cell apoptosis and inflammation. MiR-93-5p served as a target of lncRNA *NORAD* gene, and its overexpression reversed the role of lncRNA *NORAD* gene in the biological function of HUVECs. The target genes of miR-93-5p were enriched in HIF-1 signaling, TGF-beta signaling and PI3K-Akt signaling, protein-protein interaction (PPI) network indicated STAT3, MAPK1 to be the key targets.

**Conclusions:**

Upregulation of expression of lncRNA *NORAD* gene was a potential diagnostic biomarker for DVT and related to the development of PTS. LncRNA NORAD/miR-93-5p axis was involved in the progress of DVT through regulating endothelial cell function.

**Supplementary Information:**

The online version contains supplementary material available at 10.1186/s12959-023-00575-3.

## Background

Deep venous thrombosis (DVT) is the common clinical cardiovascular disease characterized by a thrombus in the lumen of a deep vein of legs [1]. Clinically, the stage of DVT can be divided into acute phase (within 14 days of onset), the subacute phase (15–30 days of onset) and the chronic phase (after 30 days of onset) [2]. Historically, DVT is diagnosed based on the primarily imaging modalities, including duplex ultrasound, helical CT scans, and venography [3]. Currently, D-dimer has been adopted in the diagnosis of DVT, but it can only be used as rule in marker for ultrasound investigations for DVT [3]. At present, the common treatment means include anti-coagulation, thrombolysis and surgical thrombectomy [4]. Post-thrombotic syndrome (PTS) is the most common sequela of DVT, occurring in up to 40% of DVT cases [5]. PTS is featured by swelling, pain, edema, venous ectasia, and skin induration of the affected limb [6]. Thereby, timely diagnosis and effective treatment are very important for the prognosis of DVT patients.

In recent years, non-coding RNAs have gradually become a hot topic in medical research, especially microRNA (miRNA), long non-coding RNA (lncRNA) and circulating RNA (circRNA). LncRNAs are widely present in various human tissues and have a wide range of gene regulatory functions. In recent years, a large number of studies have shown that lncRNA has regulatory effects on cardiovascular diseases, malignant tumors, nervous system diseases, endocrine diseases, etc. [7]. With the improvement of detection methods, the discovery of serum lncRNA provides a new class of biomarkers for the diagnosis of a variety of diseases [7]. Dysregulation of the expression of lncRNAs has been detected in DVT patients, such as nuclear enriched abundant transcript 1 (NEAT1) and X inactive specific transcript (XIST) [8, 9]. In fact, a number of lncRNAs have been identified to be closely related to endothelial injury, which is the major risk factor for the progress of DVT [10, 11]. For example, elevated expression of lncRNA *XIST* gene was detected in the plasma of DVT patients, the activity restriction and apoptosis of human umbilical vein endothelial cells (HUVECs) caused by lncRNA *XIST* gene are the mechanisms involved in DVT progression [9]. The results indicate that these dysregulated expression of lncRNAs have the potential as a marker of DVT, and are involved in the pathogenesis of disease.

The non-coding RNA activated by DNA damage (NORAD) is a novel lncRNA, its role in vascular endothelial cell injury, atherosclerosis, and coronary artery disease has been recently presented [12]. In a study about atherosclerosis (AS), lncRNA *NORAD* gene was determined to be at high expression in both oxidized low-density lipoprotein (ox-LDL) induced HUVECs and high fat diet (HFD)-treated mice, lncRNA *NORAD* gene knockdown was suggested to relieve vascular endothelial cell injury [12]. In another in vivo study, increased NORAD was identified in atherosclerotic mouse aortas, which was related to inflammation, oxidative stress and endothelial dysfunction in atherosclerotic mouse aortas [13]. In consideration of the important role of in the pathogenesis of DVT, the role of lncRNA *NORAD* gene in the development of DVT attracted our attention. Therefore, the present study was designed to determine the expression changes and contribution of lncRNA *NORAD* gene in the diagnosis for acute DVT patients. Moreover, the prognostic value of lncRNA *NORAD* gene in the occurrence of PTS was examined based on the follow-up results. In addition, the possible mechanism of lncRNA *NORAD* gene in the development of DVT was further explored in vitro.

## Methods

### Study population

85 cases with acute lower extremity DVT were enrolled in the present study who admitted to Taihe Hospital, Affiliated Hospital of Hubei University of Medicine from January 2021 to December 2022. Another 85 cases who were suspected DVT but actually DVT-negative according to the Duplex ultrasound examination were selected as the control group. This study was conducted under the supervision and approval of the Ethics Committee of Taihe Hospital, Affiliated Hospital of Hubei University of Medicine.

Inclusion criteria of DVT cases: (1) aged from 18 to 65 years old; (2) patients were informed and voluntarily agreed to participate in the study; (3) patients had clinical symptoms such as swelling and pain in the affected limb; (4) central or whole-limb DVT was diagnosed based on the lower extremity venous Duplex ultrasound examination; (5) time of onset was less than 14 days; (6) all patients were unilateral first disease. Exclusion criteria: (1) poor body tolerance; (2) patients who cannot undergo catheterization thrombolytic therapy; (3) there is coagulation dysfunction; (4) patient was complicated with mental illness and could not cooperate to complete this study. All patients were followed up for 18 months to record the development of PTS. PTS was scored according to the International Society on Thrombosis Haemostasis (ISTH) guidance, patients were diagnosed with PTS if the Villalta score was at least 5 [14].

### Demographics and laboratory data

Demographic data including age, gender, body mass index (BMI), medical history (such as hypertension, diabetes mellitus, hyperlipidemia), and smoking habits were recorded in hospitalization. The blood test was done using an automatic CBC analysis device (Beckman Coulter Inc., CA, USA) for the collection of laboratory data within 24 h of admission. Blood samples were initially centrifuged at 1000× g for 15 min and the resulted supernatant was centrifuged again at 2500× g for 15 min for serum sample collection. The serum samples were transferred into frozen tubes and stored at − 80 °C.

### Cell culture and transfection

HUVECs were gained from the American Typical Culture Conservatory (ATCC), which were cultured in DMEM maintained in a 37 °C, 5% CO_2_ incubator. 10% fetal bovine serum and 10% endothelial cell growth supplement were added to the medium. Small interfering (si) RNA (si-NORAD) sequence of lncRNA *NORAD* gene and its negative control (si-NC), and overexpression plasmid of pcDNA3.1-NORAD were provided by GenePharma Co, while miR-93-5p mimic, mimic-NC, miR-93-5p inhibitor and inhibitor-NC were obtained from the RIBOBIO Co. When cells grew to 80% confluence, the cell transfection was performed using Lipofectamine 3000 (Invitrogen, USA). After 5 h of culture, a new medium was replaced.

### RNA extraction and real-time quantitative reverse transcription PCR (RT-qPCR)

Clinical serum samples and HUVECs were used for the total RNA extraction using Trizol LS reagent (Invitrogen, USA). After purity identification, RNA reverse transcription was performed by Fastking gDNA Dispelling RT SuperMix Kit or miRcute Plus miRNA First Stand cDNA Kit (TIANGEN, Beijing, China). SuperReal PreMix Plus (SYBR Green) or miRcute Plus miRNA qPCR Kit (SYBR Green, TIANGEN, Beijing, China) were applied for qPCR on ABI PRISM 7300 (ABI)., while the primer sequences were designed and synthesized by RIBOBIO Co. The relative expression of NORAD and miR-93-5p was calculated by 2^−ΔΔCt^ method. GAPDH was used as the internal reference of lncRNA *NORAD* gene while U6 was for the internal reference of miR-93-5p, because they were stably expressed and proven to be suitable internal controls. Relative quantities of gene expression levels were normalized to the reference genes (GAPDH or U6) and then normalized to the control group.

### Cell proliferation

100 µl HUVECs resuspension was plated into a 96-well plate. Daily cell viability detection was performed for three days consecutively. 10 µl cell counting kit-8 (CCK-8, Dojindo, Japan) was added to the well and incubated for 2 h. The optical density (OD) at 450 nm was tested.

### Cell apoptosis

After cell transfection, cells in each group were collected to detect the apoptotic rate using Annexin V-FITC Apoptosis Detection Kit. Specifically, HUVECs were resuspended and incubated with 5 µl Annexin V-FITC mixed with 5 µl propidium iodide (PI) for 5 min in the dark. Finally, the cell apoptotic rate was detected on a flow cytometer.

### Cell migration

Cell migration was measured using Trabswell (Corning, USA). The resuspended HUVECs were seeded into the upper chamber of the Transwell, while the lower chamber was filled with 500 µl of DMEM. After incubation for 24 h, the cells migrated into the lower chamber were stained with methanol and crystal violet. After a water rinse, the migrated cells were counted under a light microscope.

### Enzyme-linked immunosorbent assay (ELISA)

The concentration of inflammatory cytokines including (TNF)-α, IL (interleukin)-6 (IL-6), and IL-1β were quantified by a commercially available ELISA Kit from Abcam (USA). In brief, the diluted samples and protein standards were added to the plate, and incubated with HRP-coupled detection antibodies. Then the termination solution was added followed by detection of absorbance at 450 nm.

### Nuclear/cytoplasmic fractionation assay

The nuclear/cytoplasmic fractionation assay was done using the commercial PARIS kit (Invitrogen, USA). The collected HUVECs were incubated with 450 µl cell grading buffer on ice. After centrifugation at 1200 rpm for 5 min, the upper cytoplasmic RNA was collected. The precipitate fraction was collected and added to the NER regent. After centrifugation, the nuclear RNA was collected. Then RT-qPCR was performed for the detection of OIP5-AS1 expression in the nucleus and cytoplasm via using U6 and GAPDH as the internal control.

### Prediction and verification of target binding

The target miRNAs with potential binding sites for NORAD were predicted through LncBook 2.0 and Starbase 3.0 (ENCORI) software. GSE173461 dataset was applied for the extraction of miRNAs related to DVT [15], and key miRNAs were identified by the overlapping in both LncBook 2.0 (https://ngdc.cncb.ac.cn/lncbook) and Starbase 3.0 ((http://starbase.sysu.edu.cn/) datasets. Prediction of miR-93-5p was performed based on TargetScan (http://www.targetscan.org/vert_72/), miRDB (http://www.mirdb.org/) and GeneCard database (http://www.genecards.org/). Then Venn diagram was performed in the online analysis toll Bioinformatics (www.bioinformatics.com) to show the number of differentially expressed genes in the database.

### Luciferase reporter assay

The target fragments containing wild type (WT) or mutant (MT) binding sites in the 3’UTR of NORAD were amplified by PCR and subcloned into the psiCHECK2 luciferase reporter vector. Then luciferase reporter assay was performed in HUVECs, and the above plasmid was co-transfected with miR-93-5p mimic or miR-93-5p inhibitor using Lipofectamine 2000, respectively. After culturation for 48 h, the cells were lysed and luciferase activity was detected through the luciferase reporting system kit (Promega, USA).

### Bioinformatics analysis

The overlapping target genes were mapped into the protein-protein interaction (PPI) networks via STRING, and the highest-confidence interaction score of more than 0.9 was used as the cutoff. Then Gene ontology (GO) analysis and Kyoto Encyclopedia of Genes and Genomes (KEGG) pathway enrichment analysis were done for the gene function annotation and pathway enrichment.

### Statistical analysis

Three independently repeated experiments were done for each assay. Data were checked for normality via the Kolmogorov–Smirnov (K-S) normality test. The continuous variables with normal distribution were presented as mean and standard deviation (SD), which were compared between groups via applying student’s *t*-test or one-way ANOVA. All data analyses were completed on SPSS 23.0 or Graphpad prism 7.0 software. The receiver operator characteristic (ROC) curve was drawn for the diagnostic value analysis of lncRNA *NORAD* gene in DVT, and the area under the curve (AUC) was calculated for the evaluation of the diagnostic potential. Moreover, cox regression analysis and Kaplan-Meier were deployed for the prognostic value assessment. *P* < 0.05 was set as the cutoff value of significance.

## Results

### Demographic and laboratory data of the subjects

The clinical basic information of the study groups was presented in Table 1. No significant difference appeared in terms of age, gender, BMI, hypertension, diabetes mellitus, hyperlipidemia and smoking (*P* > 0.05). Almost all coagulation indicators including prothrombin time (PT), antithrombin (AT), international normalized ratio (INR), fibrinogen (FIB) and D-dimer significantly differed from the case and control groups (*P* < 0.05), except for thrombin time (TT). For DVT patients, their duration symptoms before diagnosis, varicose veins at diagnosis and DVT location were also recorded in Table 1.

**Table 1:** Baseline information of the study objects

Variables	Control group (n = 85)	DVT group (n = 85)	*P* value
Age, year	49.44 ± 6.84	49.98 ± 7.05	0.612
Gender, n (%)			0.167
Male	37 (43.53)	46 (54.12)	
Female	48 (56.47)	39 (45.88)	
BMI, n (%)			0.524
≥ 25 kg/m^2^	33 (38.82)	29 (34.12)	
< 25 kg/m^2^	52 (61.18)	56 (65.88)	
Hypertension, n (%)			0.607
Yes	22 (25.88)	25 (29.41)	
No	63 (74.12)	60 (70.59)	
Diabetes mellitus, n (%)			0.599
Yes	7 (8.24)	9 (10.59)	
No	78 (91.76)	76 (89.41)	
Hyperlipidemia, n (%)			0.679
Yes	15 (17.65)	13 (15.29)	
No	70 (82.35)	72 (84.71)	
Smoking, n (%)			0.167
Yes	27 (31.76)	19 (22.35)	
No	58 (68.24)	66 (77.65)	
Coagulation indicators, n (%)			
PT, s	17.96 ± 2.99	8.65 ± 1.26	< 0.001
AT, µg/mL	125.06 ± 11.69	103.79 ± 9.01	< 0.001
TT, s	20.19 ± 5.02	20.39 ± 4.36	0.782
INR	0.97 ± 0.21	2.28 ± 0.57	< 0.001
FIB, g/L	3.22 ± 0.61	5.34 ± 0.80	< 0.001
D-dimer, ng/mL	131.54 ± 9.28	180.02 ± 13.71	< 0.001
Duration symptoms before diagnosis, weeks			-
≥ 2	-	19 (22.35)	
< 2	-	66 (77.65)	
Varicose veins at diagnosis, n (%)			-
Yes	-	27 (31.76)	
No	-	58 (68.24)	
Localization of DVT, n (%)			-
Calf vein	-	13 (15.29)	
Popliteal vein	-	33 (38.83)	
Femoral and iliac vein	-	39 (45.88)	

PT, prothrombin time; AT, antithrombin; TT, thrombin time; INR, international normalized ratio; FIB, fibrinogen

### Expression change and diagnostic value assessment of serum NORAD in DVT patients

**Fig. 1:**
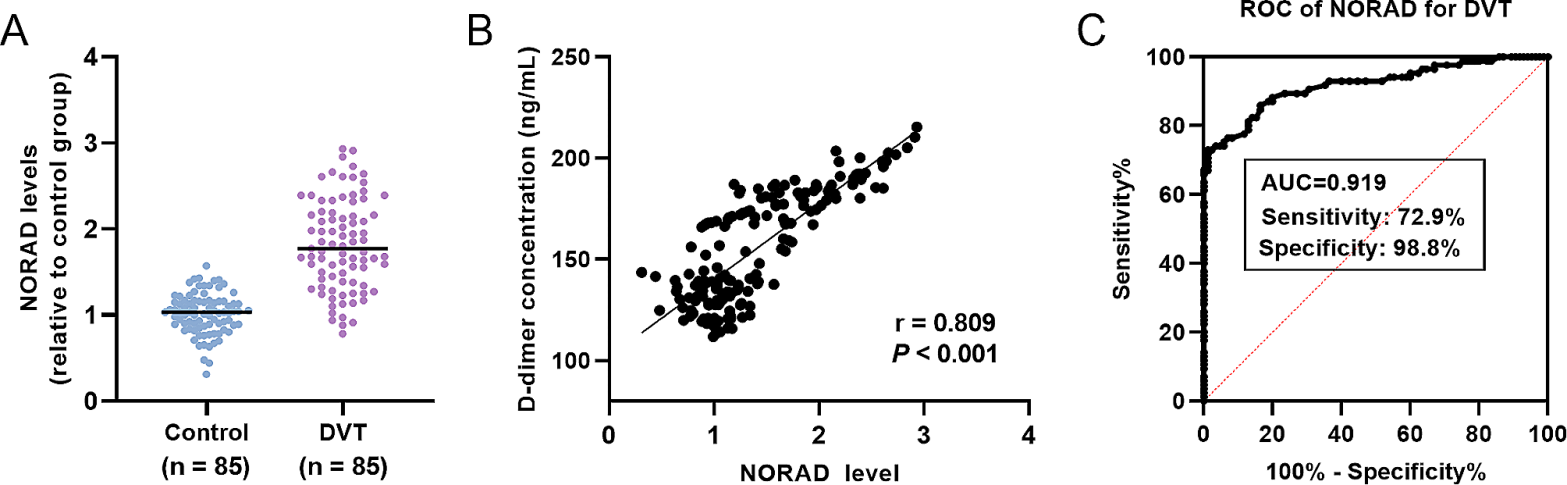
Expression changes and clinical value of lncRNA *NORAD* gene in DVT. (**A**) High serum NORAD levels in the serum of DVT patients. Data were expressed as mean and SD. **** *P* < 0.0001. (**B**) Expression of lncRNA *NORAD* gene showed prominent correlation with D-dimer concentration in all participants. (**C**) ROC curve of lncRNA *NORAD* gene for distinguishing DVT

As shown by qRT-PCR analysis results, lncRNA *NORAD* gene was at high expression in the serum of DVT patients (Fig. 1A, *P* < 0.001). In addition, the correlation of lncRNA *NORAD* gene with D-dimer concentration was also evaluated. Based on the Pearson’s correlation analysis results, the expression of lncRNA *NORAD* gene showed a prominent correlation with D-dimer concentration in all participants (r = 0.809, *P* < 0.001, Fig. 1B). Moreover, its diagnostic potential was evaluated through drawing ROC curve (Fig. 1C). It was observed that the expression of lncRNA *NORAD* gene can distinguish DVT patients from healthy controls, with the AUC of 0.919. The maximum value of the Yoden index was 0.718 with the cutoff value at 1.44. The sensitivity and specificity were 72.90% and 98.8%, respectively.

### Clinical value of NORAD in predicting PTS onset of DVT patients

**Fig. 2:**
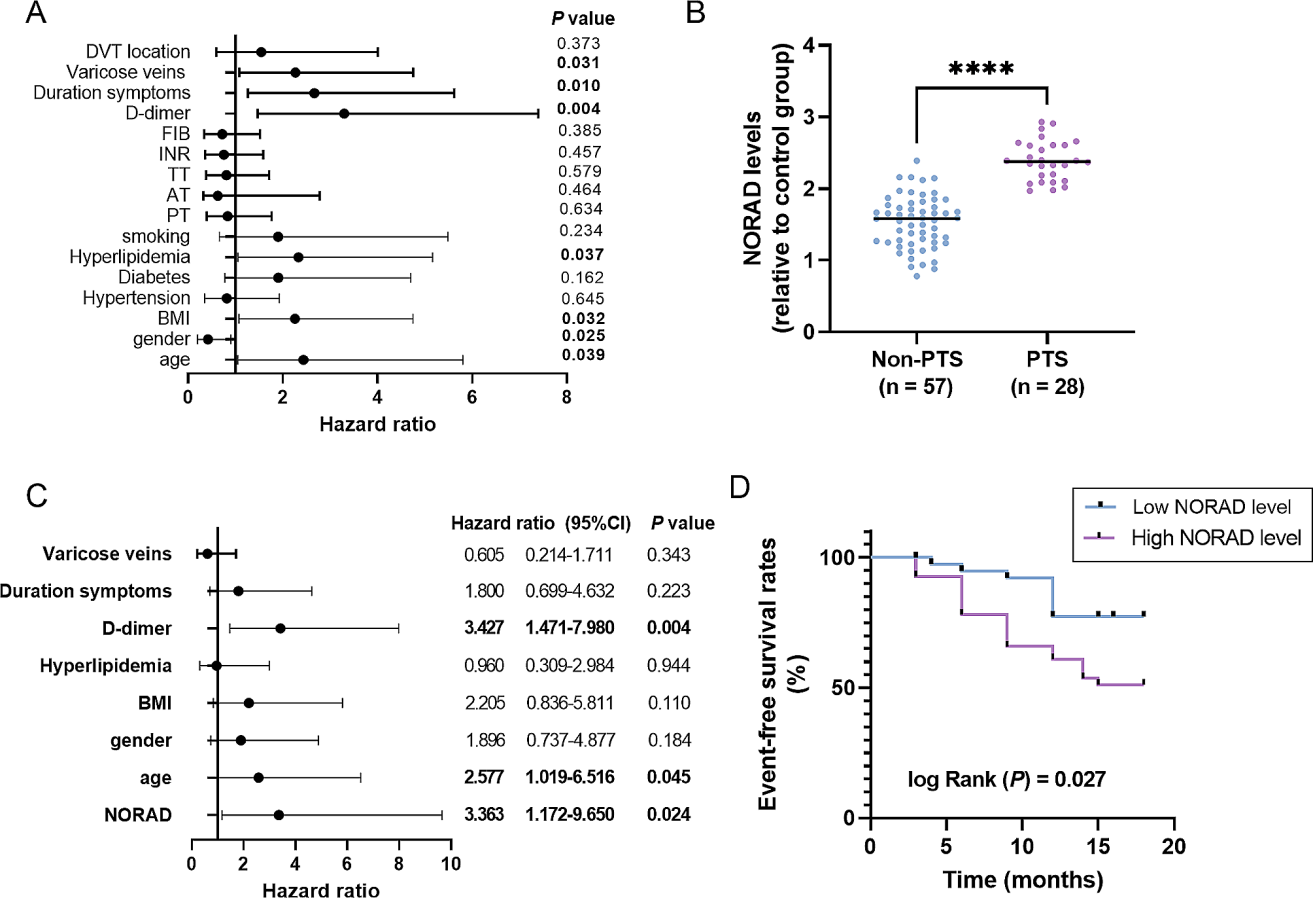
Clinical value of NORAD in predicting PTS. (**A**) Univariate cox regression analysis to assess the association of clinical indicators with PTS. (**B**) Expression of lncRNA *NORAD* gene in the serum of PTS cases. **** *P* < 0.0001. (**C**) Cox regression analysis indicates independent influence factors related to the onset of PTS. (**D**) Kaplan-Meier plot of lncRNA *NORAD* gene in the onset PTS

According to the follow-up information, a total of 28 cases developed into PTS during 18 months follow-up time. Based on the univariate cox regression analysis results, a total of seven clinical indicators presented significant levels, namely varicose veins, duration symptoms, D-dimer, hyperlipidemia, BMI, gender and age (Fig. 2A, *P* < 0.05). These seven indicators that showed significant level were further introduced into the multiple Cox regression analysis model. Interestingly, qRT-PCR results indicated the elevated expression of lncRNA *NORAD* gene in PTS patients (Fig. 2B), which was also introduced in the multiple Cox regression analysis. As displayed in Fig. 2C, D-dimer (HR = 3.427, 95% CI = 1.471–7.980), age (HR = 2.577, 95% CI = 1.019–6.516) and lncRNA *NORAD* gene (HR = 3.363, 95% CI = 1.172–9.650) were independently influence factors related to the development of PTS for DVT patients. Moreover, Kaplan-Meier plot was drawn to more intuitively present the predictive value of lncRNA *NORAD* gene in PTS (Fig. 2D). It was seen that DVT cases with high levels of lncRNA *NORAD* gene were more susceptible to PTS (*P* = 0.027).

### Role verification of NORAD in cell functions of HUVECs

**Fig. 3:**
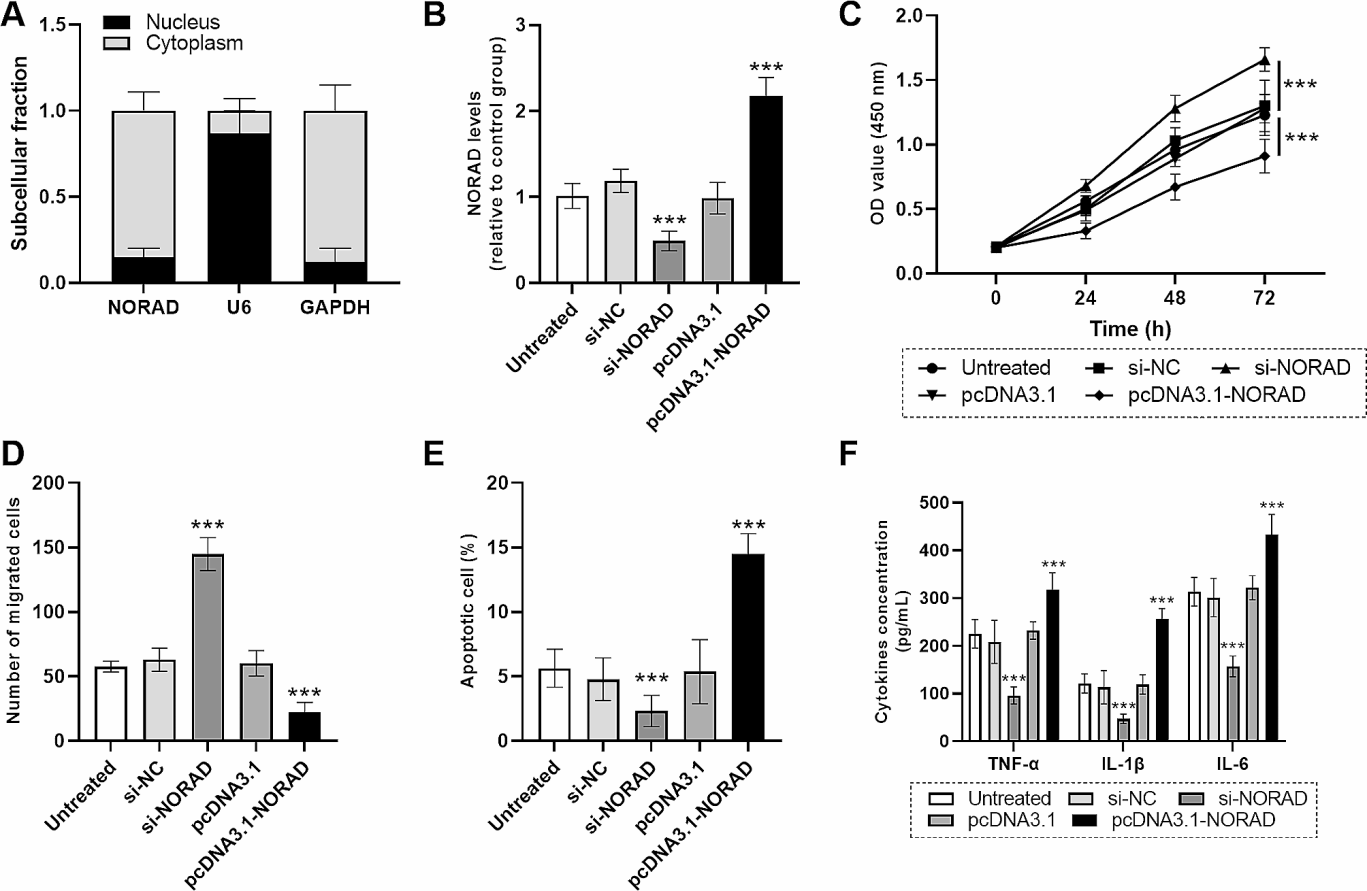
Role of lncRNA *NORAD* gene in cell functions of HUVECs. (**A**) Subcellular localization of lncRNA *NORAD* gene in HUVECs. (**B**) Expression changes of lncRNA *NORAD* gene in HUVECs after cell transfection. (**C**) CCK-8 results of HUVECs after cell transfection. (**D**) Number of migrated cells after cell transfection. (**E**) Apoptotic cell percentage after cell transfection. (**F**) Concentration of inflammatory cytokines in HUVECs after cell transfection. Data were expressed as mean and SD. *** means *P* < 0.001

To excavate the potential mechanisms of NORAD in DVT, its subcellular localization was accomplished and lncRNA *NORAD* gene was found to be mainly located in the cytoplasm of HUVECs (Fig. 3A). Levels of lncRNA *NORAD* gene were mediated via cell transfection to investigate its role in DVT. As displayed in Fig. 3B, si-NORAD transfection contributed to the downregulation of lncRNA *NORAD* gene while pcDNA3.1-NORAD led to the levels’ upregulation (*P* < 0.001). Besides, the function experiments determined that lncRNA *NORAD* gene knockdown promoted HUVECs’ proliferation, migration while suppressing cell apoptosis, but lncRNA *NORAD* gene upregulation brought an adverse effect (Fig. 3C–E). Similarly, inflammatory cytokines namely TNF-α, IL-1β and IL-6 were also remarkably diminished by lncRNA *NORAD* gene knockdown, which was aggravated by lncRNA *NORAD* gene upregulation (Fig. 3F).

### Target miRNAs of lncRNA NORAD gene

**Fig. 4:**
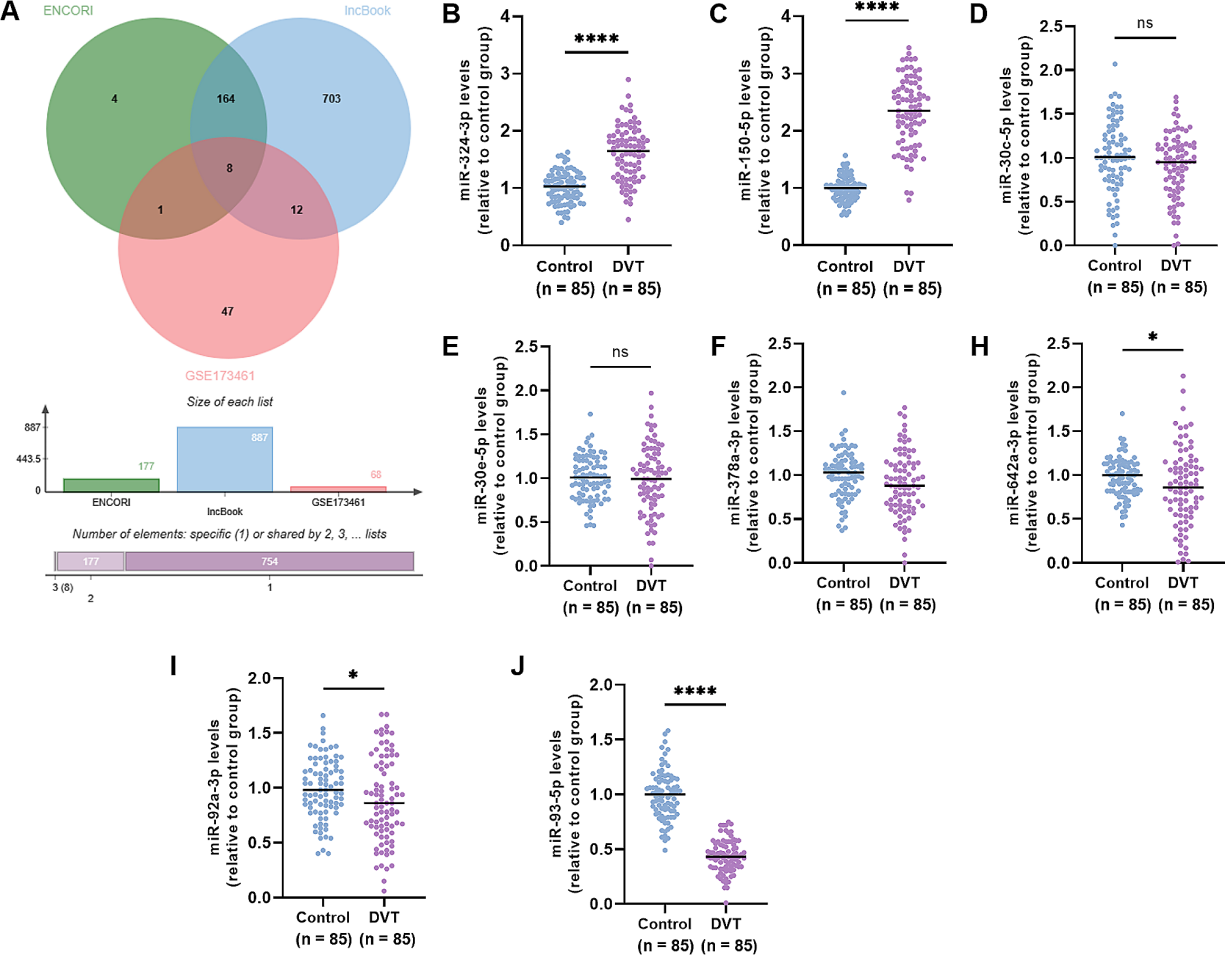
Target miRNAs of lncRNA *NORAD* gene. (**A**) Overlapping target miRNAs of lncRNA *NORAD* gene in LncBook 2.0 and Starbase 3.0 (ENCORI) datasets. SE173461 dataset was used for the extraction of miRNAs related to DVT that overlapped in both LncBook 2.0 and Starbase 3.0 datasets. (**B–J**) Levels of eight overlapping miRNAs in the serum of DVT patients calculated by qRT-PCR. Data were expressed as mean and SD. * *P* < 0.05; **** *P* < 0.0001

The predicted target miRNAs with potential binding sites for lncRNA *NORAD* gene were obtained through LncBook 2.0 and Starbase 3.0 (ENCORI) software. As shown in Fig. 4A, 172 miRNAs were predicted. Moreover, GSE173461 dataset was used for the extraction of miRNAs related to DVT that overlapped in both LncBook 2.0 and Starbase 3.0 datasets. Finally, a total of 8 miRNAs were identified, including miR-324-3p, miR-150-5p, miR-30c-5p, miR-30e-5p, miR-378a-3p, miR-642a-3p, miR-92a-3p, miR-93-5p. Furthermore, the expression changes of these night miRNAs were verified in the clinical serum samples. Among the eight miRNAs, only two were significantly upregulated in the serum of DVT patients, namely miR-324-3p and miR-150-5p (Fig. 4B, C, *P* < 0.001). The expression changes of miR-30c-5p, miR-30e-5p and miR-378a-3p were not significant between the case and control groups (Fig. 4D–F,* P* > 0.05). As displayed in Fig. 4H–J, miR-642a-3p, miR-92a-3p, miR-93-5p were all significantly downregulated in DVT patients, in which miR-93-5p expression changes were the most significant (*P* < 0.001). Thus miR-93-5p was selected for further mechanism exploration.

### NORAD serves as competing endogenous RNA (ceRNA) of miR-93-5p

**Fig. 5:**
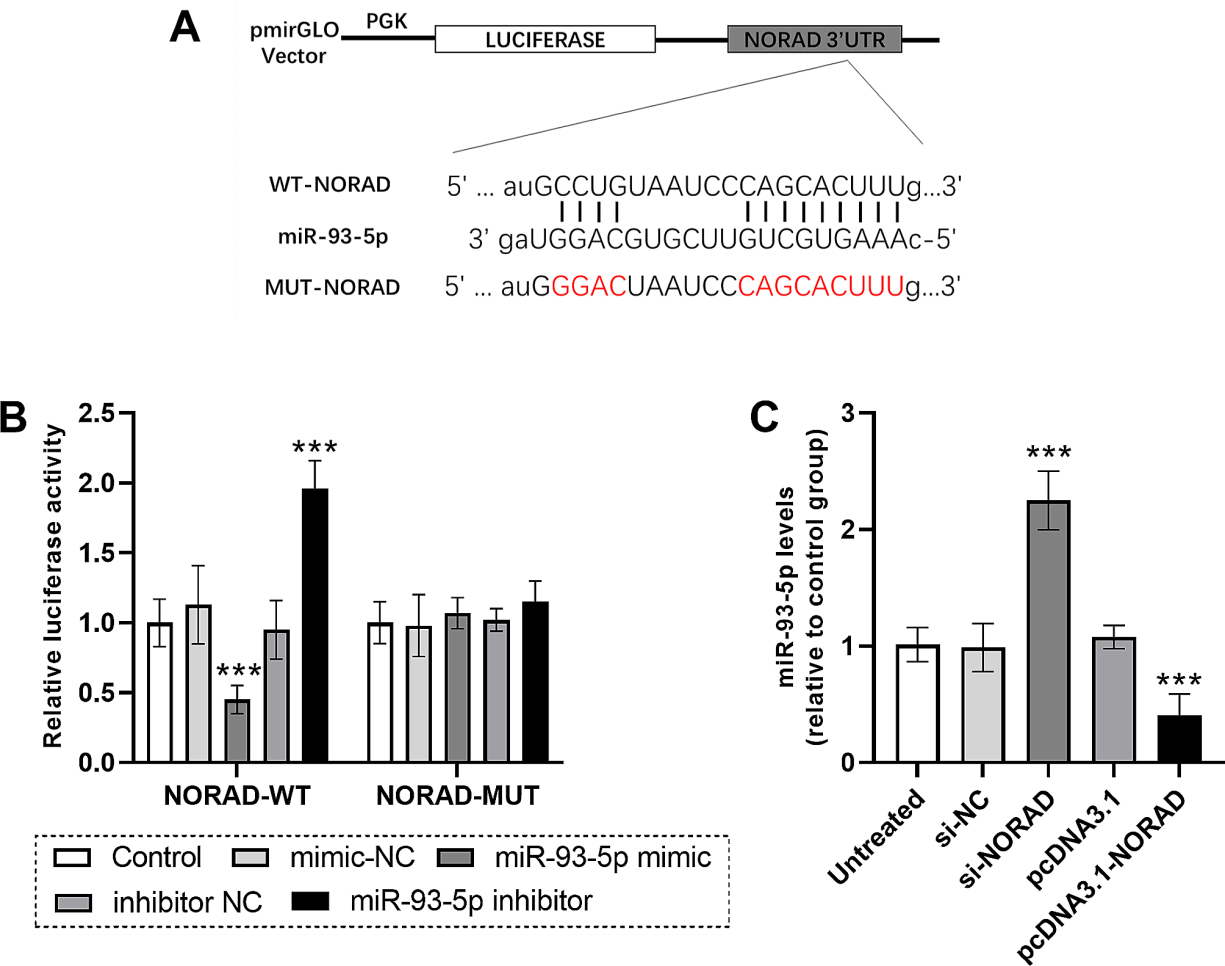
LncRNA *NORAD* gene serves as ceRNA competitively binds miR-93-5p. (**A**) The target binding sites of lncRNA *NORAD* gene with miR-93-5p. (**B**) Luciferase activity of cells transfected with different sequence. (**C**) MiR-93-5p levels in HUVECs after mediating expression of lncRNA *NORAD* gene. Data were expressed as mean and SD. *** *P* < 0.001 compared with the control/untreated group

Target miRNAs of lncRNA *NORAD* gene was subsequently explored. Figure 5A shows the target binding sites of lncRNA *NORAD* gene with miR-93-5p. The miR-93-5p mimic typically decreased the luciferase activity of NORAD-WT, while the miR-93-5p inhibitor significantly increased the luciferase activity of lncRNA *NORAD* gene (*P* < 0.05, Fig. 5B), but neither of them had a significant effect on the luciferase activity of NORAD-MUT (*P* > 0.05, Fig. 5B). In HUVECs, si-NORAD transfection was accompanied with the upregulation of miR-93-5p, while lncRNA *NORAD* gene upregulation led to the downregulation of miR-93-5p (Fig. 5C).

### MiR-93-5p reversed the role of NORAD in biological function of HUVECs

**Fig. 6:**
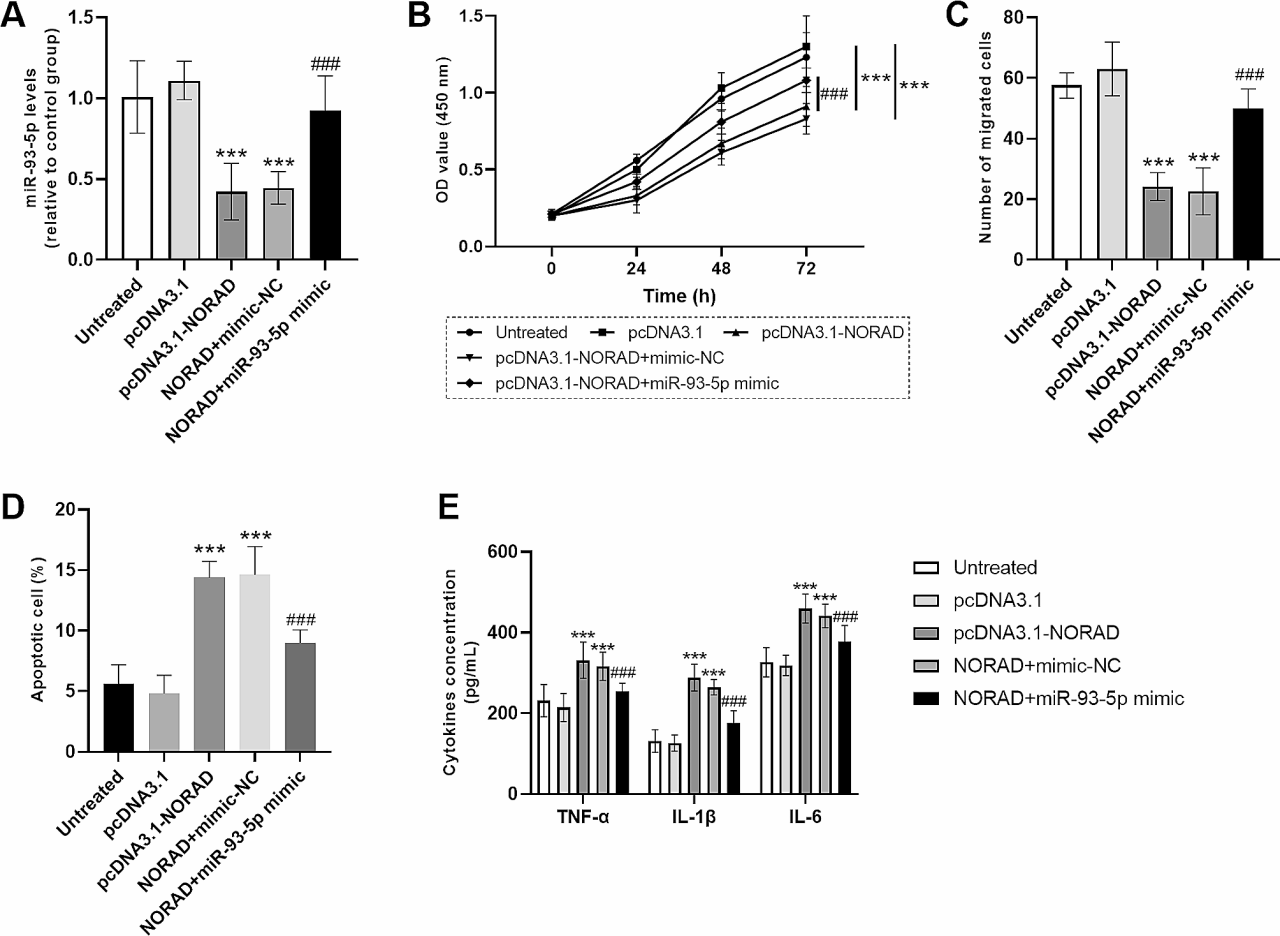
miR-93-5p reversed the role of lncRNA *NORAD* gene in biological function of HUVECs. (**A**) miR-93-5p levels in HUVECs after cell transfection. (**B**) Cell viability of HUVECs after cell transfection. (**C**) Cell migration of HUVECs after cell transfection. (**D**) Cell apoptosis of HUVECs after cell transfection. (**E**) Concentrations of inflammatory cytokines in HUVECs after cell transfection. Data were expressed as mean and SD. *** *P* < 0.001 compared with the untreated group; ### *P* < 0.001 compared with pcDNA3.1-NORAD group

The co-regulatory function of lncRNA *NORAD* gene and miR-93-5p was distinguished in HUVECs. As seen in Fig. 6A, pcDNA3.1-NORAD transfection decreased the levels of miR-93-5p, which was greatly restored by miR-93-5p mimic transfection (Fig. 6A). In addition, lncRNA *NORAD* gene upregulation-induced inhibition of cell viability and migration was also reversed by miR-93-5p overexpression (Fig. 6B, C). Similarly, the promotion of cell apoptosis induced by lncRNA *NORAD* gene was also restored by miR-93-5p (Fig. 6D). What’s more, lncRNA *NORAD* gene stimulated the release of inflammatory cytokines, including TNF-α, IL-1β and IL-6. But the influence was back-spined by miR-93-5p (Fig. 6E).

### Functional and pathway enrichment analysis of predicted target genes of miR-93-5p by GO and KEGG

**Fig. 7:**
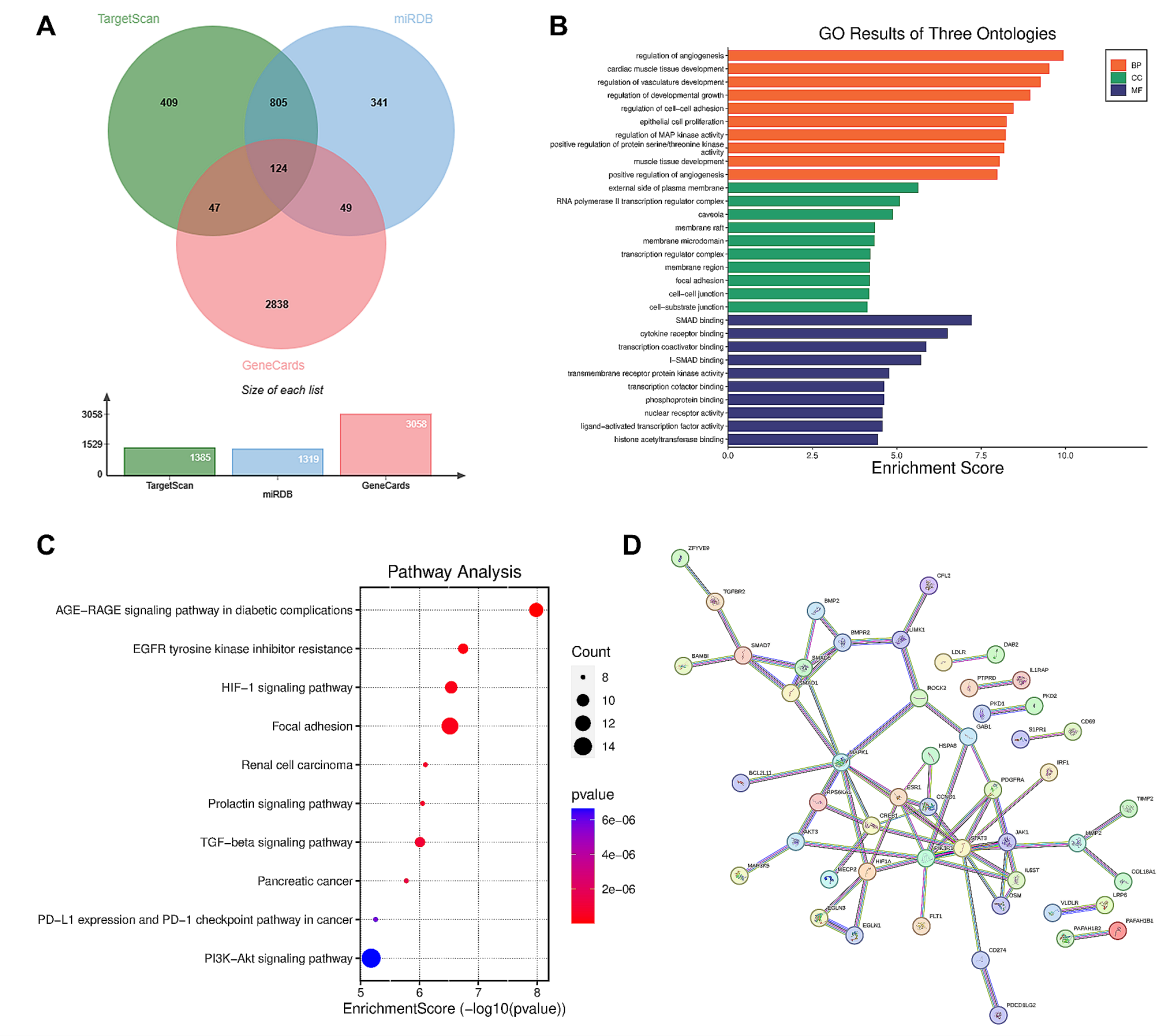
Functional and pathway enrichment analysis of predicted target genes of miR-93-5p. (**A**) Venn diagram of miR-93-5p target genes based on TargetScan, miRDB and GeneCard database. (**B**) Go results of biological process (BP), cellular component (CC), and molecular function (MF). (**C**) Pathway enrichment results by KEGG. (**D**) PPI networks among target genes mapped in STRING

Prediction of miR-93-5p was performed based on TargetScan, miRDB and GeneCard database. As the Venn diagram shown, a total of 124 genes were overlapped (Fig. 7A). GO terms enabled the function enrichment analysis, including three items of biological process (BP), cellular component (CC), and molecular function (MF). As seen in Fig. 7B, the target genes are mainly enriched in regulation of angiogenesis, cardiac muscle tissue development, external side of plasma membrane, RNA polymerase II transcription regulator complex, SMAD binding, cytokine receptor binding and so on. Furthermore, based on the KEGG analysis, HIF-1 signaling, TGF-beta signaling and PI3K-Akt signaling were mainly enriched (Fig. 7C).

Moreover, the PPI networks among target genes were mapped in STRING. As seen in Fig. 7D, a PPI network of 124 nodes and 68 edges was constructed, with the *P* value less than e^− 16^. The top 10 hub genes were presented in Supplementary Table based on the degree of connectivity, namely STAT3, MAPK1, PIK3R1, ESR1, CREB1, HIF1A, JAK1, SMAD5, BMPR2 and CCND1.

## Discussion

Deep vein thrombosis (DVT) is a common peripheral vascular disease in clinical practice, with an annual incidence of 0.1–0.27% [16]. The physiological basis of DVT includes vascular endothelium damage, slow blood flow velocity and blood hypercoagulability [17]. Recent studies have supported that lncRNA has regulatory effects on cell proliferation, migration, apoptosis and other functions [16]. Moreover, the regulatory role of lncRNAs in vascular endothelial injury is almost certain [16]. LncRNA *NORAD* gene is a vascular endothelial cell injury-related lncRNA. In AS studies, lncRNA *NORAD* gene was indicated to stimulate vascular endothelial cell injury through mediating cell inflammation and oxidative stress [12, 13]. Based on the present qRT-PCR results, lncRNA *NORAD* gene was determined to be at high expression in the serum of DVT patients. Clinically, serum levels of lncRNA *NORAD* gene can distinguish DVT patients from healthy controls. The findings indicated the diagnostic potential of lncRNA *NORAD* gene in DVT. It is known that PTS is a long-term complication of DVT, and the incidence of PTS within 2 years is 20–50%, even if DVT patients receive standardized anticoagulant therapy [18]. In the present study, all DVT patients were followed up for 18 months to record the occurrence of PTS. It can be seen that approximately 33% of patients developed into PTS, which was consistent with the previous reported incidence [19]. Moreover, qRT-PCR results indicated the elevated expression of lncRNA *NORAD* gene levels in serum of PTS patients. Based on the multiple Cox regression analysis results, lncRNA *NORAD* gene was independently related to the development of PTS during the follow-up time. Collectively, serum expression of lncRNA *NORAD* gene had considerable diagnostic and prognostic significance in discriminating DVT patients and healthy people. In addition, our present results also indicated the close relationship of age and D-dimer with the development of PTS, which was consistent with the previous evidence [20, 21].

Vascular endothelial injury is one of the important mechanisms of DVT [22]. Thus, HUVECs were applied for the cell function experiments. It was found that lncRNA *NORAD* gene knockdown promoted HUVECs’ proliferation, migration while suppressing cell apoptosis. Consistently, under hypoxic conditions, lncRNA *NORAD* gene knockdown was determined to promote HUVECs’ migration and tube formation [23]. In ox-LDL- induced HUVECs models, lncRNA *NORAD* gene knockdown contributed to the recovery of cell apoptosis induced by ox-LDL [12]. It is known that vascular endothelial cell inflammation contributes to the development of DVT [24]. Based on our present cell experiment results, inflammatory cytokines including TNF-α, IL-1β and IL-6 were also remarkably diminished by lncRNA *NORAD* gene knockdown in HUVECs. Collectively, it was concluded that lncRNA *NORAD* gene knockdown may protect against DVT through improving vascular endothelial injury and inflammation.

MiRNAs serve as targets of lncRNAs that degrade or inhibit the translation of target genes [25]. In DVT, the dysregulation and involvement of miRNAs have been widely reported [26, 27]. In the current study, miR-93-5p was identified to the the candidate target of NORAD in DVT based on bioinformatic analysis and luciferase reporter assay. Moreover, in clinical serum samples, downregulated miR-93-5p was detected in DVT patients. Thus, its involvement in the role of lncRNA *NORAD* gene was explored in HUVECs via cell transfection. The findings indicated that miR-93-5p reversed the role of lncRNA *NORAD* gene in biological function of HUVECs, lncRNA *NORAD* gene serves as ceRNA of miR-93-5p in DVT. Consistently, in mice models with myocarditis, the development of cardiac microvascular endothelial injury was accompanied by the downregulation of miR-93-5p [28]. The further rescue experiment results indicated that the upregulation of miR-93-5p can alleviate cardiac microvascular endothelial injury via suppressing inflammatory response [28]. In the sepsis-induced acute kidney injury mouse model, the involvement of miR-93-5p was determined to be associated with the endothelial protection of the endothelial progenitor cell (EPC)-derived extracellular vesicles [29]. All evidence supported the crucial role of miR-93-5p in DVT.

Accumulating evidence demonstrates that miRNAs and lncRNAs interact to regulate target genes and further mediate the downstream signaling pathways and thus modulate disease progression [27]. Therefore, the target genes of miR-93-5p were predicted and subsequently enriched by the GO and KEGG. According to the GO analysis results, the functions were mainly enriched in angiogenesis, cardiac muscle tissue development, external side of plasma membrane, RNA polymerase II transcription regulator complex, SMAD binding, cytokine receptor binding and so on. As reported, angiogenesis is the key component of DVT resolution and restitution of vascular patency after thrombosis [30, 31]. It was concluded that lncRNA *NORAD* gene played an important role in angiogenesis in function, which might be its protective mechanism in DVT and followed PTS. In addition, HIF-1 signaling, TGF-beta signaling and PI3K-Akt signaling were enriched by KEGG analysis. HIF-1 pathway was known to stimulate coagulopathy and recruitment of inflammatory cytokines, which was related to the onset and development of DVT [32, 33]. In the development of DVT, overexpression of TGF-β was detected, indicating its important role in disease progression [34]. The role of TGF-β signaling pathway in venous calcification was reported, and venous calcification was a further adverse progression after PTS [35]. In addition, TGF-β signaling was determined to be involved in the occurrence of thrombophlebitis [36]. The phosphoinositide 3-kinase (PI3K)/AKT signaling pathway was an important contributor in inflammatory response [37]. In DVT mice, the activation of PI3K/AKT signaling was determined, which was related to endothelial cell injury and inflammation [38]. These findings explained the possible involvement mechanism of NORAD/miR-93-5p in the progress of DVT. Moreover, PPI network indicated STAT3, MAPK1 to be the key targets, which were all involved in the development of DVT [39].

Although the current findings provide the direction for our later research on the mechanism of lncRNA *NORAD* gene in DVT, the key genes and downstream signaling pathways did not verified. In addition, an external validation of expression of lncRNA *NORAD* gene in a DVT cohort was necessary, which was a limitation of our study. The present findings should be verified in another study population with larger sample size. Besides, we used ISTH guidance for PTS diagnosis, but there was no clear guidelines and diagnostic criteria for PTS currently. So the role of lncRNA *NORAD* gene in PTS required further verification. Moreover, in vivo studies need to be considered to validate the role of lncRNA *NORAD* gene in DVT today.

## Conclusions

In summary, upregulation of lncRNA *NORAD* gene was identified to be a potential diagnostic biomarker for DVT, moreover, it is related to the development of PTS. In vitro, lncRNA *NORAD* gene may aggravate the vascular endothelial injury via sponging miR-93-5p. The present findings expand new perspectives on the diagnosis and mechanism exploration of DVT.

### Electronic supplementary material

Below is the link to the electronic supplementary material.

**Supplementary Material 1: Supplementary Table**. The top 10 nodes of PPI network.

## Data Availability

The datasets used and/or analysed during the current study are available from the corresponding author on reasonable request.

## Abbreviations

ATCC: American Typical Culture Conservatory
AUC: Area under the curve
BP: Biological process
CC: Cellular component
circRNA: Circulating RNA
DVT: Deep venous thrombosis
ELISA: Enzyme-Linked immunosorbent assay
EPC: Endothelial progenitor cell
GO: Gene ontology
KEGG: Kyoto Encyclopedia of Genes and Genomes
lncRNA: Long non-coding RNA
MF: Molecular function
miRNA: MicroRNA
MT: Mutant
PI: Propidium iodide
PI3K: Phosphoinositide 3-kinase
PTS: Post-thrombotic syndrome
ROC: Receiver operator characteristic
RT-qPCR: Real-time quantitative reverse transcription PCR
SD: Standard deviation
si-NC: Negative control siRNA
si-NORAD: NORAD small interfering (si) RNA
WT: Wild type
HUVECs: Human umbilical vein endothelial cells
