# Supplementary material for: Diagnostic and prognostic potential of long non-coding RNA NORAD in patients with acute deep vein thrombosis and its role in endothelial cell function
Source: Thromb J. 2024 Jan 2;22:3. doi: 10.1186/s12959-023-00575-3 (PMC10763087; doi:10.1186/s12959-023-00575-3)
Supplement: Supplementary file 1 — Supplementary Material 1: Supplementary Table. The top 10 nodes of PPI network. [file 12959_2023_575_MOESM1_ESM.docx]

**Supplementary Table** The top 10 nodes of PPI network

| Node | Identifier | Degree |
| --- | --- | --- |
| STAT3 | 9606.ENSP00000264657 | 13 |
| MAPK1 | 9606.ENSP00000215832 | 10 |
| PIK3R1 | 9606.ENSP00000428056 | 8 |
| ESR1 | 9606.ENSP00000405330 | 6 |
| CREB1 | 9606.ENSP00000387699 | 5 |
| HIF1A | 9606.ENSP00000437955 | 5 |
| JAK1 | 9606.ENSP00000499900 | 5 |
| SMAD5 | 9606.ENSP00000441954 | 5 |
| BMPR2 | 9606.ENSP00000363708 | 4 |
| CCND1 | 9606.ENSP00000227507 | 4 |
